# Enhanced visualization of microbiome data in repeated measures designs

**DOI:** 10.3389/fgene.2024.1480972

**Published:** 2024-11-15

**Authors:** Amarise Little, Rebecca A. Deek, Angela Zhang, Ni Zhao, Wodan Ling, Michael C. Wu

**Affiliations:** ^1^ Public Health Sciences Division, Fred Hutchinson Cancer Center, Seattle, WA, United States; ^2^ Department of Biostatistics, University of Pittsburgh, Pittsburgh, PA, United States; ^3^ Department of Biostatistics, University of Washington, Seattle, WA, United States; ^4^ Department of Biostatistics, Johns Hopkins University, Baltimore, MD, United States; ^5^ Division of Biostatistics, Department of Population Health Sciences, Weill Cornell Medicine, New York, NY, United States

**Keywords:** longitudinal, temporal, microbiome, PCoA, visualization method, kernel matrix

## Abstract

**Introduction:**

Repeated measures microbiome studies, including longitudinal and clustered designs, offer valuable insights into the dynamics of microbial communities and their associations with various health outcomes. However, visualizing such multivariate data poses significant challenges, particularly in distinguishing meaningful biological patterns from noise introduced by covariates and the complexities of repeated measures.

**Methods:**

In this study, we propose a framework to enhance the visualization of repeated measures microbiome data using Principal Coordinates Analysis (PCoA) adjusted for covariates through linear mixed models (LMM). Our method adjusts for confounding variables and accounts for the repeated measures structure of the data, enabling clearer identification of microbial community variations across time points or clusters.

**Results:**

We demonstrate the utility of our approach through simulated scenarios and real datasets, showing that it effectively mitigates the influence of nuisance covariates and highlights key axes of microbiome variation.

**Discussion:**

This refined visualization technique provides a robust tool for researchers to explore and understand microbial community dynamics in repeated measures microbiome studies.

## 1 Introduction

Cross-sectional studies of the human microbiome have identified a wide range of outcomes and exposures associated with the human microbiome ranging from type 2 diabetes (Qin et al., 2012; Larsen et al., 2010) to colorectal cancer (Zackular et al., 2014; Feng et al., 2015). These findings have shed light on the biological mechanisms underlying many conditions and provided critical clues as to novel therapeutic and risk reduction interventions, but a serious weakness of many of these classical studies is the limited information on how the microbiome changes over time and how these changes may affect outcomes. This has motivated the development of longitudinal microbiome studies where specimens are collected and profiled over a period of time. These studies promise comprehensive opportunities to gain better insight into topics such as how the microbiome varies over the course of a treatment (Lawrence et al., 2014; Jackson et al., 2016) and how the microbiome may sit within the causal pathway of some conditions (Turnbaugh et al., 2006; Wang et al., 2018), among many other opportunities. However, despite the potential and promises, serious analytical challenges persist.

Visualization is a particular challenge in longitudinal microbiome studies, yet it represents an important and standard step in most microbiome data analysis workflows. As in the analysis of other omics data types, visualization allows for the assessment of outlying samples and quality control. However, within the context of microbiome studies, it takes on an additional role by allowing for an understanding of microbial community structures in relation to metadata. The most popular approach for visualizing microbiome data is through Principal Coordinates Analysis (PCoA) (Gower, 2014) in which individual samples are plotted on a scatter plot. Like classical principal component analysis (PCA), the coordinates of each sample are determined based on capturing the greatest variability in the data, but whereas PCA focuses on linear transformations, PCoA allows for better capture of nonlinear transformations. Operationally, PCoA is performed by calculating a matrix of pair-wise dissimilarity between samples, where the dissimilarity measure is thought to be ecologically relevant and captures certain aspects of community structure (e.g., phylogeny, presence/absence, and abundance of taxa, etc.). The dissimilarity matrix is then transformed to a matrix of pair-wise similarity through Gower centering, and the principal coordinates are calculated as the eigenvectors of the similarity matrix. Such analyses have enabled a better understanding of many conditions and even the discovery of possible enterotypes (clusters) (The Human Microbiome Project Consortium, 2012; Arumugam et al., 2011).

In longitudinal microbiome studies, PCoA could provide information on how community structures change over time and how these changes correlate with variables of interest. However, guidance on how to carry out PCoA in longitudinal studies including multiple samples from the same subjects is lacking. PCoA strategies for repeated measures, such as in longitudinal studies, face additional challenges due to the inherent correlation between repeated observations from the same subjects. Currently, data across all subjects and time points are amalgamated, and plots are generated in the same way as in cross-sectional studies. This approach, however, may not be suitable when the goal is to analyze the temporal dynamics or subject-specific effects.

In longitudinal studies, factors such as subject clustering, irrelevant variables, and time effects often dominate the variability, overshadowing structures related to the variables of interest. Applying PCoA without adjustments for repeated measures may obscure important temporal patterns or subject-level effects, as the method assumes that all samples are independent. For example, high correlation between samples from the same subject can distort shifts over time or distort relationships with other variables.

How to accommodate these obfuscating effects in longitudinal analysis remains unclear. Our proposed method accounts for repeated measures by incorporating random effects and covariates to model within-subject correlations. This approach yields more accurate visualizations of temporal changes and effects of interest, ensuring that the primary sources of variability reflect the variables of interest rather than confounding factors.

For longitudinal microbiome studies, we propose a strategy to mitigate the effects of variables and data characteristics that may obscure the primary structures of interest in PCoA plots. This approach is also applicable to other repeated measures study designs, such as clustered microbiome measurements from individuals sharing households. Therefore, throughout this discussion, we will refer to repeated measures designs more broadly. Our proposed framework involves removing the confounding effects from the pair-wise similarity matrix while accommodating the repeated-measure nature of the data. Our framework is similar to covariate-adjusted PCoA (Shi et al., 2020), which is used for cross-sectional studies, but it also accounts for correlation among observations by incorporating random effects in a linear mixed model (LMM). Specifically, we estimate the similarity matrix, adjust out potential obfuscating effects from each PC of the similarity matrix using LMMs, and reconstruct the similarity matrix for usual PCoA analysis using the residuals from the LMM. Since there are multiple notions of residuals in LMMs (Verbeke et al., 1997), we consider multiple concepts of residuals (including marginal and standardized residuals) and provide guidance on their recommended use.

We find that our approach effectively distills the most important axes of microbiome community variation while reducing the influence of nuisance covariates in our visualizations, all while leveraging shared information across distinct time points. However, we also found that relying on marginal or conditional residuals from LMMs can be inadequate for certain analytic objectives, as unwanted structures may persist. This persistence is due to dependencies introduced by the replacement of parameters with their estimates, a challenge that is particularly pronounced in dependent data (Wakefield, 2013). To overcome these challenges, we further recommend standardization of residuals as an essential step to mitigate dependencies when the dependencies are not of primary interest. Collectively, we find that our approach and recommendations offer the ability to facilitate understanding of shifts in community structure in relation to time or other variables of interest.

In the following sections, we first review PCoA before describing our proposed strategies for adjusting out uninteresting effects. We then apply our approach within simulated examples to illustrate the potential utility and to offer specific guidance. Finally, we demonstrate the utility of our approach in two real data sets before concluding with a brief discussion.

## 2 Methods

In this section, we briefly review the usual PCoA and covariate adjusted PCoA for cross-sectional studies before presenting our proposed strategy for accommodating repeated measures study designs. We then discuss the simulation setup for some illustrative scenarios.

### 2.1 PCoA and adjusted PCoA for cross-sectional studies

Consider a cross-sectional (single time point) microbiome profiling study in which the abundances of p taxa have been quantified across n independent samples. Then n×p matrix $Y=\left[Y^{\left(1\right)},Y^{\left(2\right)},\cdots ,Y^{\left(p\right)}\right]$ , where $Y^{\left(k\right)}$ represents the vector of observations for the $k^{\text{th}}$ taxon in the study. Since visualization of multivariate data is challenging when p is large, the objective of PCoA is to find a lower dimensional representation of the data such that the most information is retained.

PCoA begins by constructing a matrix of pair-wise dissimilarities between each pair of samples, D , where the ${\left(i,i^{\prime }\right)}^{\text{th}}$ element of D is the dissimilarity between samples i and $i^{\prime }$ . A wide range of, typically nonlinear and ecologically relevant, dissimilarities are commonly used within the microbiome analysis literature. Each dissimilarity emphasizes different aspects of the data and captures different qualities of the community structure. For example, Bray-Curtis dissimilarity (Roger Bray and Curtis, 1957) emphasizes relative abundance such that more common taxa tend to drive dissimilarity. On the other hand, UniFrac distance (Lozupone and Knight, 2005) focuses on dissimilarity based on the presence/absence of taxa such that rarer taxa can exert greater influence, and further incorporates phylogenetic relationships among taxa when estimating distance. Extensions of UniFrac, such as weighted UniFrac (Lozupone et al., 2007) and generalized UniFrac (Chen et al., 2012), focus on more common taxa or compromise between rare and common taxa, respectively, while still accommodating phylogeny. Other distances such as Aitchison distance (Aitchison, 1982) try to respect compositional effects. The choice of distance is based on the type of relationships that investigators seek to study or believe to be relevant.

After calculation of D , the distance is transformed to an n×n similarity matrix which we denote K via Gower centering:

$$K=\left(I-H\right)D^{2}\left(I-H\right)$$

where $H=1{\left(1^{\prime }1\right)}^{-1}1^{\prime }$ . The principal coordinates (PCs) are computed as the eigenvectors of K , and the proportion of variability in the nonlinear space explained by each PC is given by the relative magnitude of the corresponding eigenvalue.

The PCs represent lower-dimensional embeddings of the data. For visualization, the top PCs are plotted against each other to generate PCoA plots. Specifically, the PCoA plot is a scatter plot where each point corresponds to a separate sample, and the coordinates of each point (sample) are determined by the value of the PCs. In practice, only the top few (sometimes only the top two) PCs are used for visualization, even though the proportion of variability explained may be modest.

A limitation of PCoA is that the PC directions can be driven by confounding variables, denoted X , which leads to the obfuscation of structures of primary interest, i.e., related to a variable of primary interest. Thus, adjusted PCoA (aPCoA) (Shi et al., 2020) was developed to address this by essentially regressing out the effect of obscuring variables. Specifically, the covariate adjusted similarity matrix is calculated as

$$K^{\mathrm{adj}}=\left(I-X{\left(X^{\prime }X\right)}^{-1}X^{\prime }\right)K\left(I-X{\left(X^{\prime }X\right)}^{-1}X^{\prime }\right).$$ (1)

Essentially, one is re-centering K based on the covariates.

One way of justifying this approach is the following. We first calculate the matrix K as before and then decompose $K=\Phi \Phi^{\prime }$ where $\Phi =\left[\Phi^{\left(1\right)},\ldots ,\Phi^{\left(n\right)}\right]=\sqrt{K}$ is a matrix square root. A natural choice of decomposition is to set $\Phi =U\Lambda^{1/2}$ where $U\Lambda U^{\prime }$ is the eigendecomposition of K . The $\Phi^{\left(k\right)}$ ’s (the columns of Φ ) are simply the original PCs. Then aPCoA essentially regresses each PC on X and calculates the residual (i.e., corrected PCs). The matrix of residuals is E with $k^{th}$ column given as

$$E^{\left(k\right)}=\Phi^{\left(k\right)}-X{\hat{\beta }}^{\left(k\right)}\text{ and }{\hat{\beta }}^{\left(k\right)}={\left(X^{\prime }X\right)}^{-1}X^{\prime }\Phi^{\left(k\right)}.$$ (2)

By regressing out the effect of the covariates X , each $E^{\left(k\right)}$ is now orthogonal to the covariates. Then the covariate-adjusted similarity matrix can be recalculated as $K^{*}=EE^{\prime }$ — note that one cannot simply use E as the PC since proportions of variability explained have shifted. At this point, PCoA proceeds as before, just using $K^{*}$ instead of K . This gives the same result as in Equation 1.

Importantly, however, although aPCoA is useful for removing covariate effects, it cannot directly handle repeated measures designs. This approach assumes that each sample is independent, which is not the case for repeated measures data. In such data, samples taken from the same subject over time are correlated, and failing to account for this correlation can result in misleading visualizations where the effects of repeated measures dominate the patterns of interest.

In contrast, our method, detailed in the following section, extends traditional aPCoA by incorporating linear mixed models (LMMs) that account for both fixed effects (e.g., treatment, age) and random effects (e.g., subject-level variability) to properly adjust for the repeated measures structure. By adjusting for within-subject correlations, this method ensures that temporal changes in microbiome profiles are more accurately captured, and that variability due to repeated measures does not obscure key patterns related to covariates or treatment effects.

### 2.2 PCoA strategy for repeated measures

As before, we assume that we have profiled p taxa for n subjects or clusters. However, we now assume that we have collected measurements $m_{i}$ times for the $i^{\text{th}}$ subject or cluster, such that all microbiome measurements are then in an M×p matrix, $Y=\left[Y^{\left(1\right)},Y^{\left(2\right)},\cdots ,Y^{\left(p\right)}\right]$ where $Y^{\left(k\right)}={\left(y_{1,1}^{\left(k\right)},\ldots ,y_{1,m_{1}}^{\left(k\right)},\ldots ,y_{n,1}^{\left(k\right)},\ldots ,y_{n,m_{n}}^{\left(k\right)}\right)}^{\prime }$ , where $y_{i,j}^{\left(k\right)}$ is the count of the $k^{\text{th}}$ taxon for subject/cluster i at measurement j and $M=\sum_{i}m_{i}$ . We assume that subjects/clusters are independent of each other but that there may be correlation within each subject/cluster.

As earlier, the goal is to adjust Y in such a way that we can meaningfully plot patterns embedded in the data. The general strategy follows a similar approach to aPCoA but includes some key differences. First, to accommodate the repeated measures and longitudinal sampling, we incorporate linear mixed models into the calculation of residuals in Equation 2. Second, because of using mixed models, the convenient formulas in Equation 1 are no longer usable in the repeated measures setting, which requires more explicit correction. Finally, there is no unique concept of “residual” for linear mixed models (Verbeke et al., 1997). With multiple notions of residual, the choice of which one to use depends on the context and analytic objective, but we suggest the use of standardized residuals in general.

**FIGURE 1:**
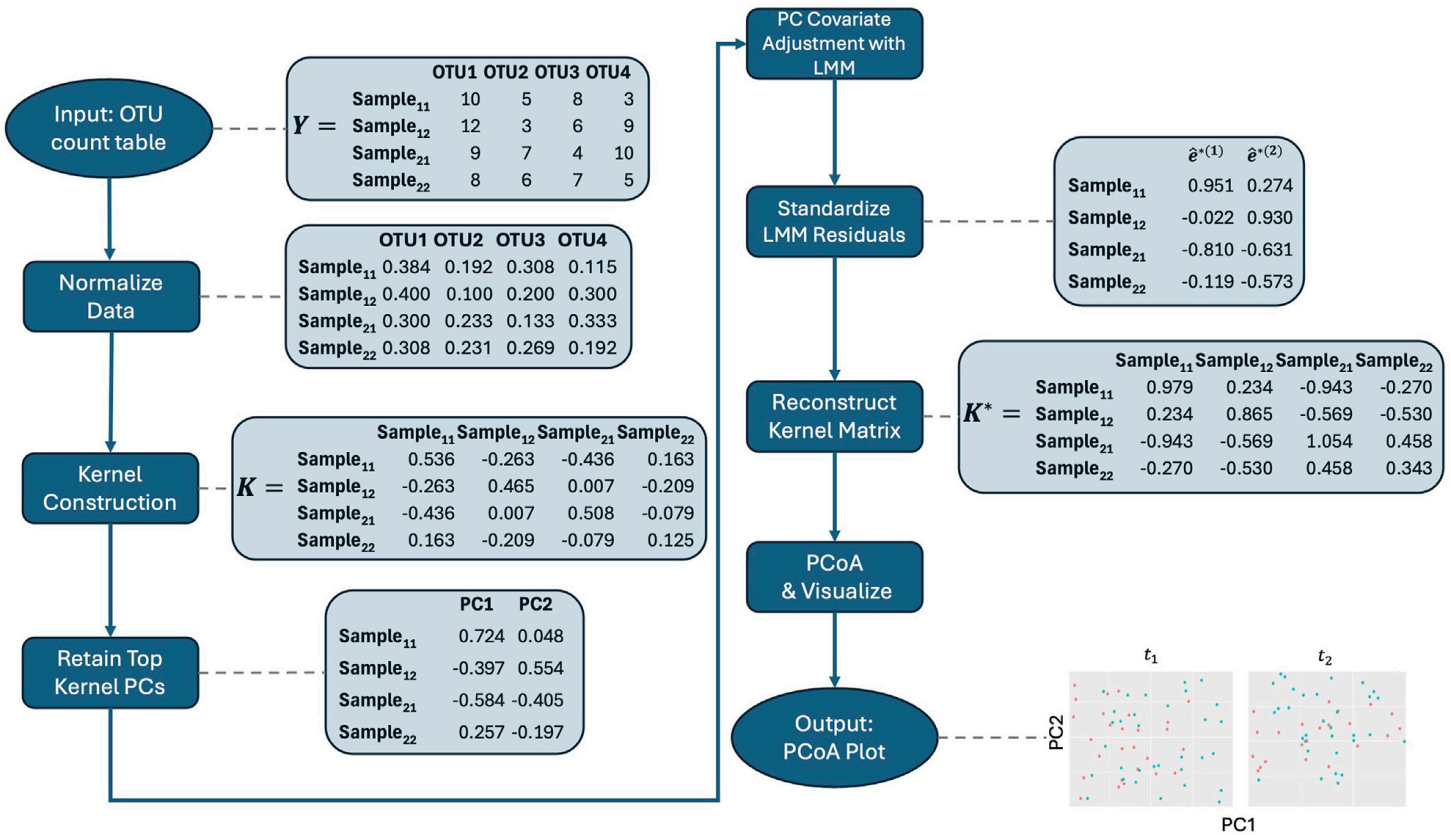
Workflow for the adjusted PCoA method applied to repeated measures microbiome data. The process starts with data normalization, followed by kernel construction. Kernel principal coordinates are extracted and adjusted for covariates using linear mixed models. The residuals are standardized, and the adjusted kernel matrix is reconstructed before visualizing the top principal coordinates using PCoA. Note that the data shown are illustrative and do not directly lead to the final plot displayed in the figure.

With these considerations in mind, we propose the following steps for our method, which are also illustrated in the workflow in Figure 1. Before applying it, we recommend normalizing the microbiome data to account for differences in library sizes across samples. Specifically, we suggest using the centered log-ratio (CLR) transformation on relative abundances in conjunction with the Aitchison kernel matrix. By expressing each feature as a log-ratio relative to the geometric mean of all relative abundances in a sample, CLR reduces the influence of total sequencing depth, making the data more comparable across samples. Additionally, CLR-transformed data are more amenable to linear transformations, which makes them particularly well-suited for methods that rely on linear modeling, such as our approach. The Aitchison kernel is ideal for compositional microbiome data, as it respects the relative nature of the data and ensures that variations in sequencing depth do not bias the results. Throughout the rest of the text, we use the Aitchison kernel for these reasons. However, if an alternative kernel matrix, such as Bray-Curtis, is needed, then relative abundance data may be more appropriate, as CLR-transformed data are not suitable for such distance measures. Therefore, users should choose between raw count, relative abundance, or CLR-transformed data based on the kernel matrix and specific analysis objectives, with the Aitchison kernel being the preferred choice for compositional data.

1. Kernel Construction: Embed microbiome data Y into an appropriate kernel (similarity) matrix K , as usual. Throughout the text, we use Aitchison kernel matrices because they are well-suited for compositional data like microbiome datasets. Specifically, Aitchison distance accounts for the relative nature of the data and is compatible with CLR-transformed data. As in earlier steps, we calculate an ecologically relevant distance between all pairs of samples, which may no longer correspond to unique individuals, and Gower-center this distance matrix to form the kernel matrix, as is typical in PCoA.
2. Kernel PCA: We then obtain kernel PCs, $\sqrt{K}=U\Lambda^{1/2}$ , from $K=U\Lambda U^{\prime }$ using all samples and without regard to the repeated measures sampling, as before.
3. Retain Key Kernel PCs: Retain the top $\ell \leq \text{rank}\left(K\right)$ kernel PCs that explain a large proportion of the variability in K . Possible choices for proportion of variability explained could be 90% or 95% .
4. Covariate Adjustment: Regress kernel PC r , r={1,2,…,ℓ} , on any “nuisance” covariates as fixed effects (e.g., age, sex, study site) and/or random effects (e.g., random slopes for time within subject/cluster), as well as random intercepts for subjects/clusters to account for repeated measurement. For subject i , given fixed effect vector $x_{i}$ and random effect vector $z_{i}$ ,

$$\Phi_{i}^{\left(r\right)}=x_{i}\beta^{\left(r\right)}+z_{i}\gamma_{i}^{\left(r\right)}+\epsilon_{i}^{\left(r\right)},$$

where $\beta^{\left(r\right)}$ is the vector of fixed effect coefficients, $\gamma_{i}^{\left(r\right)}$ is the vector of random effect coefficients for the $i^{\text{th}}$ subject/cluster, and $\epsilon_{i}^{\left(r\right)}$ is the vector of residual errors.

5. Standardize Residuals: Obtain estimated standardized residuals ${\hat{e}}^{*\left(r\right)}$ . To do so, first, obtain marginal (population-level) residuals, $e_{i}^{\left(r\right)}=\Phi_{i}^{\left(r\right)}-x_{i}{\hat{\beta }}^{\left(r\right)}$ . If $V_{i}$ is the true error structure, then $\text{var}\left(e_{i}^{\left(r\right)}\right)=V_{i}$ , and $\text{var}\left({\hat{e}}_{i}^{\left(r\right)}\right)\approx {\hat{V}}_{i}$ . The dependence within our residuals may affect visualization of the data. Therefore, we standardize to remove the dependence within the residuals. Next, we let ${\hat{V}}_{i}=L_{i}L_{i}^{\prime }$ be the Cholesky decomposition of ${\hat{V}}_{i}$ . Then, we can form estimated standardized residuals

$${\hat{e}}_{i}^{*\left(r\right)}=L_{i}^{-1}{\hat{e}}_{i}^{\left(r\right)}=L_{i}^{-1}\left(\Phi_{i}^{\left(r\right)}-x_{i}{\hat{\beta }}^{\left(r\right)}\right).$$

Now, $\text{var}\left({\hat{e}}_{i}^{*\left(r\right)}\right)\approx I$ .

6. PCoA on Reconstructed Adjusted Kernel Matrix: Then as in aPCoA, we reconstruct the adjusted kernel matrix $K^{*}$ , from which we can perform usual PCoA. For completeness, this proceeds by calculating the eigendecomposition of $K^{*}=\left(U^{*}\right)\Lambda^{*}{\left(U^{*}\right)}^{\prime }$ . The PCs are then the columns of $\sqrt{K^{*}}=U^{*}{\left(\Lambda^{*}\right)}^{1/2}$ .
7. Visualize: The top PCs, or first few columns, of $\sqrt{K^{*}}$ can be plotted against each other. The proportion of residual variability explained is given by the relative magnitude of the diagonal values of $\Lambda^{*}$ .

Note that in Step 3, we drop a few of the PCs that explain very small proportions of the variability. This step is not theoretically necessary but is important in practice as the low variability of the tail eigenvectors leads to difficulties in fitting the mixed models. Due to the low percentage of variability explained, their removal typically does not affect the overall visualization.

For supplementary analyses comparing the proposed kernel-based aPCoA and aPCoA on CLR-transformed data, please refer to the Supplementary Material.

### 2.3 Simulated scenarios

The core of our proposed strategy for PCoA with repeated measures is to adjust out the effects of variables that obscure or confound visualization. The specific variables that should be included inside of X depend on the analytic objectives. Here, we briefly consider some general guidance on what variables one may wish to include in X by studying some specific scenarios. We emphasize that these examples are not intended to be a comprehensive catalog of analytic objectives but rather provide some ideas for possible strategies.

#### 2.3.1 Time-invariant covariate obscuring the effect of another time-invariant covariate

Time-invariant covariates are variables that remain constant over time, such as treatment arm, sex, and binary smoking history. We first explore a simulated example where we aim to visualize how microbiome profiles evolve in relation to a time-invariant covariate (treatment arm, in this simulation). However, this visualization is obscured by the influence of another time-invariant covariate (sex, in this simulation). Specifically, we consider a hypothetical microbiome study where a treatment assigned at baseline affects community diversity over time for some individuals. Our goal is to visually assess whether microbiome profiles differ across treatment arms, but the influence of sex obscures this visualization, so we wish to adjust for it.

For this scenario, we simulated a longitudinal dataset with repeated measurements from n=100 subjects across $m_{i}=m=4$ time points: $\left\{t_{0},t_{1},t_{2},t_{3}\right\}$ . Subjects were randomly assigned to the treatment or control arm with equal probability. Sex (male or female) was also simulated for each subject with equal probability, independently of arm assignment. For each subject and time point, we simulated microbiome profiles using real data from the Multi-Omic Microbiome Study-Pregnancy Initiative (MOMS-PI), sourced from the HMP2Data R package. We focused on vaginal samples from site visit 4, excluding samples with a library size of fewer than 4,000 and taxa with a zero occurrence rate greater than 95% . After pre-processing, 270 samples and 233 operational taxonomic units (OTUs) remained.

Let $y_{i,j}$ denote the length p vector of taxon counts for sample i at time $t_{j}$ . We randomly selected n samples from the MOMS-PI data. For each selected sample, we perturbed the taxa counts by drawing from a Dirichlet distribution with parameters set to the MOMS-PI taxa counts plus a small pseudocount of 0.5. The resulting Dirichlet samples were then scaled by multiplying them by the sample’s original MOMS-PI library size, ensuring the perturbed counts retained the same library size. These perturbed counts represent the baseline microbiome profiles, denoted $y_{i,0}$ , i={1,2,…,n} .

To simulate taxa counts at $t_{j}$ , j=1 we perform the following steps. We first initialized taxa counts according to a multinomial distribution with number of trials equal to the subject’s library size at $t_{j-1}$ and probabilities generated from a Dirichlet distribution with concentration parameters equal to $y_{i,j-1}+0.5$ . Next, to introduce changes in the community profiles shared by all subjects, we randomly selected 20 taxa and reduced their counts by multiplying them by 0.25. For those in the treatment group, we randomly selected a different set of 20 taxa and increased their counts by a factor of 3. To introduce an obscuring sex effect, we multiplied the counts of another 20 taxa by 8 for female subjects. The sex-specific taxa were selected by identifying pairs of taxa with prevalence closest to each of 0,0.1,0.2,…,0.9 , ensuring that the sex effect spans a range of taxa prevalence. Notably, the sex effect size is significantly larger than the treatment effect size, so we expect any visualization that does not adjust for sex to be overwhelmingly driven by the sex effect.

We repeat these steps to generate taxa counts for $t_{j}$ , j={2,3} . None of the taxa sets overlap, and we used the same taxa sets across all time points. Finally, all taxonomic abundances were rounded to the nearest whole number to reflect counts.

We compared the proposed method described in Section 2.2 to the aPCoA method for cross-sectional studies, as outlined in Section 2.1, and to an approach where we only adjusted for sex as a fixed effect.

For the aPCoA approach, we first stratified all data points by time. Within each stratum, we embedded the simulated microbial counts into an Aitchison distance, generated a kernel matrix, and obtained the kernel PCs. We then regressed each kernel PC on sex in a linear model, computed the residuals, and derived the PCs from the resulting residuals matrix. Finally, we plotted PC2 against PC1 for each time point.

For the fixed-effect-only approach, we embedded all simulated microbial counts Y into an Aitchison distance, generated a kernel matrix K , and obtained the kernel PCs. We then regressed each kernel PC on sex in a linear model, computed the residuals, and derived the PCs from the resulting residuals matrix. Finally, we plotted PC2 against PC1 for each time point.

For the proposed aPCoA approach for repeated measures, we embedded the simulated microbial counts Y into an Aitchison distance, which was used to generate a kernel matrix K . We then obtained the kernel PCs (Φ) from K and retained the top ℓ=69 kernel PCs, which explained approximately 90.1% of the variability in K . Each of the ℓ kernel PCs was regressed on sex, time, and their interaction as fixed effects, and a random intercept for each subject, using a linear mixed model. The estimated standardized residuals were then computed, an adjusted kernel matrix was constructed, and the PCs of the adjusted kernel matrix were obtained. Finally, we plotted PC2 against PC1 for each time point.

#### 2.3.2 Time-varying covariate obscuring the effect of a time-invariant covariate effect

While the first example focused on two time-invariant covariates, we now consider a situation where the primary interest remains a time-invariant covariate (again, treatment). However, in this scenario, a time-varying covariate influences the microbiome profiles over time, obscuring the visualization of the time-invariant effect. A hypothetical example might involve a treatment effect of interest, but with some subjects falling ill at various points during the study. The illness impacts the microbiome profiles, but the timing of sickness varies across subjects.

For this scenario, we simulated data on n=100 subjects at m=4 time points, following a similar approach to that described in Section 2.3.1. We initialized data at $t_{0}$ the same as in the previous section. Sickness status was simulated as a time-varying binary covariate, following a standard Bernoulli distribution independently across time points. For subjects who were sick at $t_{0}$ , we multiplied the counts of 20 randomly selected sickness-affected taxa by 24.

For subsequent time points, $t_{j}$ , j∈{1,2,3} , we initialized taxa counts as in Section 2.3.1; generated shared changes in the community profiles across all subjects by multiplying the counts of 20 randomly chosen taxa by 0.25; generated a treatment effect by multiplying the counts of 20 randomly selected taxa by 3; and finally generated a sickness effect by multiplying the counts of the differentially prevalent taxa (described in the previous section) by 24. None of the taxa sets overlap, and we used the same taxa sets across all time points. After counts at all time points were generated, we rounded all taxa counts to the nearest whole number.

We compared the proposed method described in Section 2.2 to the aPCoA method for cross-sectional studies, as outlined in Section 2.1, and to an approach where we only adjusted for sickness status as a fixed effect.

For the aPCoA approach, we first stratified all data points by time. Within each stratum, we embedded the simulated microbial counts into an Aitchison distance, generated a kernel matrix, and obtained the kernel PCs. We then regressed each kernel PC on sickness status in a linear model, computed the residuals, and derived the PCs from the resulting residuals matrix. Finally, we plotted PC2 against PC1 for each time point.

For the fixed-effect-only approach, we embedded all simulated microbial counts Y into an Aitchison distance, generated a kernel matrix K , and obtained the kernel PCs. We then regressed each kernel PC on sickness status in a linear model, computed the residuals, and derived the PCs from the resulting residuals matrix. Finally, we plotted PC2 against PC1 for each time point.

For the proposed aPCoA approach for repeated measures, we embedded the simulated microbial counts Y into an Aitchison distance, which was used to generate a kernel matrix K . We then obtained the kernel PCs (Φ) from K and retained the top ℓ=62 kernel PCs, which explained approximately 90.0% of the variability in K . Each of the ℓ kernel PCs was regressed on sickness status as a fixed effect, and a random intercept for each subject, using a linear mixed model. The estimated standardized residuals were then computed, an adjusted kernel matrix was constructed, and the PCs of the adjusted kernel matrix were obtained. Finally, we plotted PC2 against PC1 for each time point.

#### 2.3.3 Hierarchical structure obscuring the effect of a time-invariant covariate effect

Here, we address a related yet distinct issue. Instead of repeated measurements arising from a longitudinal study design, repeated measures in this case result from the hierarchical structure of the data, where observations are nested within higher-level units. For example, we may have microbiome measurements from multiple members of the same households or from multiple body sites within individuals. Unlike in the previous two sections, these data are not longitudinal, but they may exhibit cluster heterogeneity that we may or may not wish to highlight in visualizations.

In this simulation, the observations are microbiome measurements from individuals within families sharing a household. Each family member undergoes a different treatment during a trial, which affects their microbiome profiles. The similarity in microbiome profiles due to household membership obscures the treatment effect we aim to visualize. Without addressing the clustering effect, data points would naturally cluster based on household membership. By effectively mitigating the clustering effect, we allow other sources of variation to emerge in the visualizations.

We simulated data for n=30 families with m=3 members in each family. For the $i^{\text{th}}$ family and the $j^{\text{th}}$ family member, $y_{i,j}$ is the vector of taxa counts. Each family member received one of three treatments, with the family member corresponding to j=1 gets treatment 1, j=2 receiving treatment 2, and so on.

We generated $y_{i,1}^{\left(k\right)}$ , i∈{1,…,n} , k∈{1,…,p} the same way we generated $y_{i,0}^{\left(k\right)}$ in Section 2.3.1. Next, we jointly sampled $y_{i,2}^{\left(k\right)}$ and $y_{i,3}^{\left(k\right)}$ , i∈{1,…,n} , k∈{1,…,p} from a multivariate normal distribution with mean ${\left(y_{i,1}^{\left(k\right)}\;y_{i,1}^{\left(k\right)}\right)}^{\prime }$ and covariance $\frac{1}{7}\left(\begin{array}{cc} 10 & 3\\ 3 & 10 \end{array}\right)$ . After generating these taxa counts, we introduced treatment effects by multiplying the counts of 20 randomly selected taxa by 8 for $y_{i,2}^{\left(k\right)}$ and by multiplying the counts of 20 different randomly selected taxa by 32 for $y_{i,3}^{\left(k\right)}$ , i∈{1,…,n} , k∈{1,…,p} . Finally, we rounded all counts to the nearest whole number, truncating any negative values to 0.

We aimed to compare visualizations that either account for or ignore the confounding effect of cluster (familial) membership on the treatment effect. We applied the standard PCoA approach as in Section 2.1, making no adjustments. We compared this to the proposed aPCoA approach for repeated measures as in Section 2.2. Following the same procedure as before, we retained the top ℓ=29 kernel PCs, which explained 88.13% of the variability. Each kernel PC was then regressed on a random intercept for each family. We then computed standardized residuals, constructed the corresponding kernel matrices, and obtained the resulting PCs.

The standard PCoA plots of PC2 vs. PC1 visualize microbiome profiles without accounting for cluster membership, while the aPCoA plots using standardized residuals account for this clustering effect.

## 3 Results

### 3.1 Results from simulated scenarios

#### 3.1.1 Time-invariant covariate obscuring the effect of another time-invariant covariate

**FIGURE 2:**
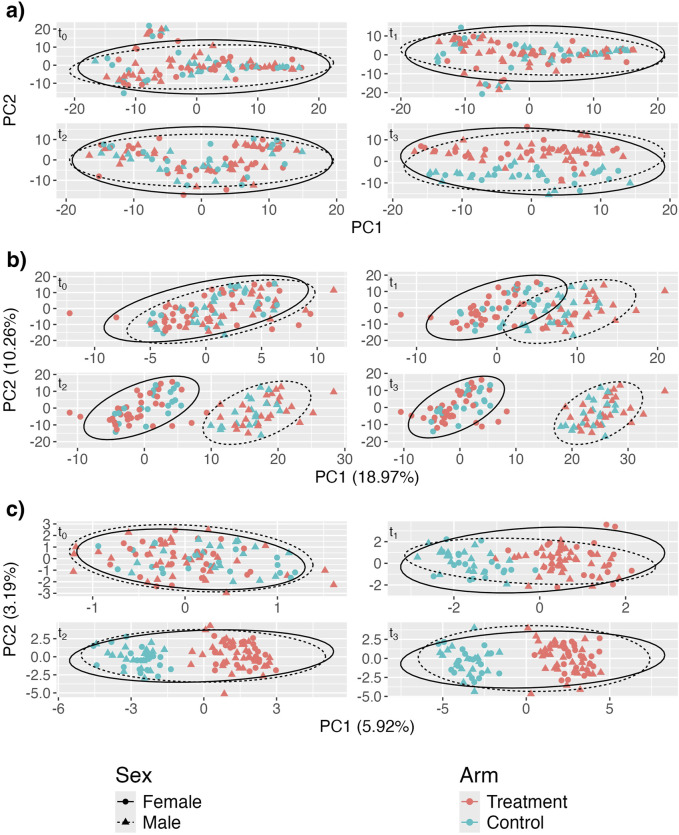
Adjusted principal coordinates analysis (aPCoA) plots of a simulated longitudinal dataset where a time-invariant covariate (sex) obscures the treatment effect ( n=100 and m=4 ). **(A)** aPCoA approach for cross-sectional data, adjusting for sex in a linear model, with data stratified by time point; **(B)** aPCoA approach adjusting only for sex in a linear model across all time points; **(C)** proposed aPCoA approach for repeated measures, adjusting for sex, time, and their interaction as fixed effects, while including a per-subject random intercept as a random effect in a linear mixed model. Points are colored by treatment arm and shaped by sex.

Figure 2A shows the aPCoA for cross-sectional data plots of microbiome profiles stratified by time point and one can see that the treatment effect is difficult to distinguish, particularly at earlier times, with the plotted points on top of each other due to the effects of sex. The difference due to treatment is not apparent until $t_{3}$ , yet from the simulation scenario, the difference should have been apparent from $t_{1}$ .

Figure 2B shows that adjusting using a linear model to account only for the fixed effect of sex is insufficient to fully remove the sex effect. While the separation between treatment and control groups is slightly more discernible compared to the unadjusted case in Figure 2A, the plots still indicate a strong influence of sex, particularly at later time points (e.g., $t_{2}$ and $t_{3}$ ). The separation between male and female subjects remains pronounced, suggesting that additional adjustments or a more complex model are necessary to adequately isolate the treatment effect from the confounding influence of sex.

Figure 2C demonstrates that the proposed aPCoA method for repeated measures successfully removes the confounding sex effect. Unlike in Figure 2B, where the sex effect dominates, in Figure 2C, the separation between treatment and control groups is clearly visible across all time points after baseline, with no clear separation of the male and female subjects. This indicates that the method effectively adjusts for the sex effect, allowing the treatment effect to emerge distinctly in the visualization. Compared to the plot in Figure 2A, the plot in Figure 2C illustrates the differences in community profiles earlier, which better reflects the true underlying model. This demonstrates the utility in borrowing information across all time points and the value of having longitudinal data. Moreover, the data points appear to segregate in a more meaningful way; treatment points migrate towards the right side of the plot as time progresses. This trend would not be guaranteed if we simply stratified microbiome profiles by time without proper adjustment, as the PCoA rotations at each time point would not necessarily be consistent with rotations at other time points.

#### 3.1.2 Time-varying covariate obscuring the effect of a time-invariant covariate effect

**FIGURE 3:**
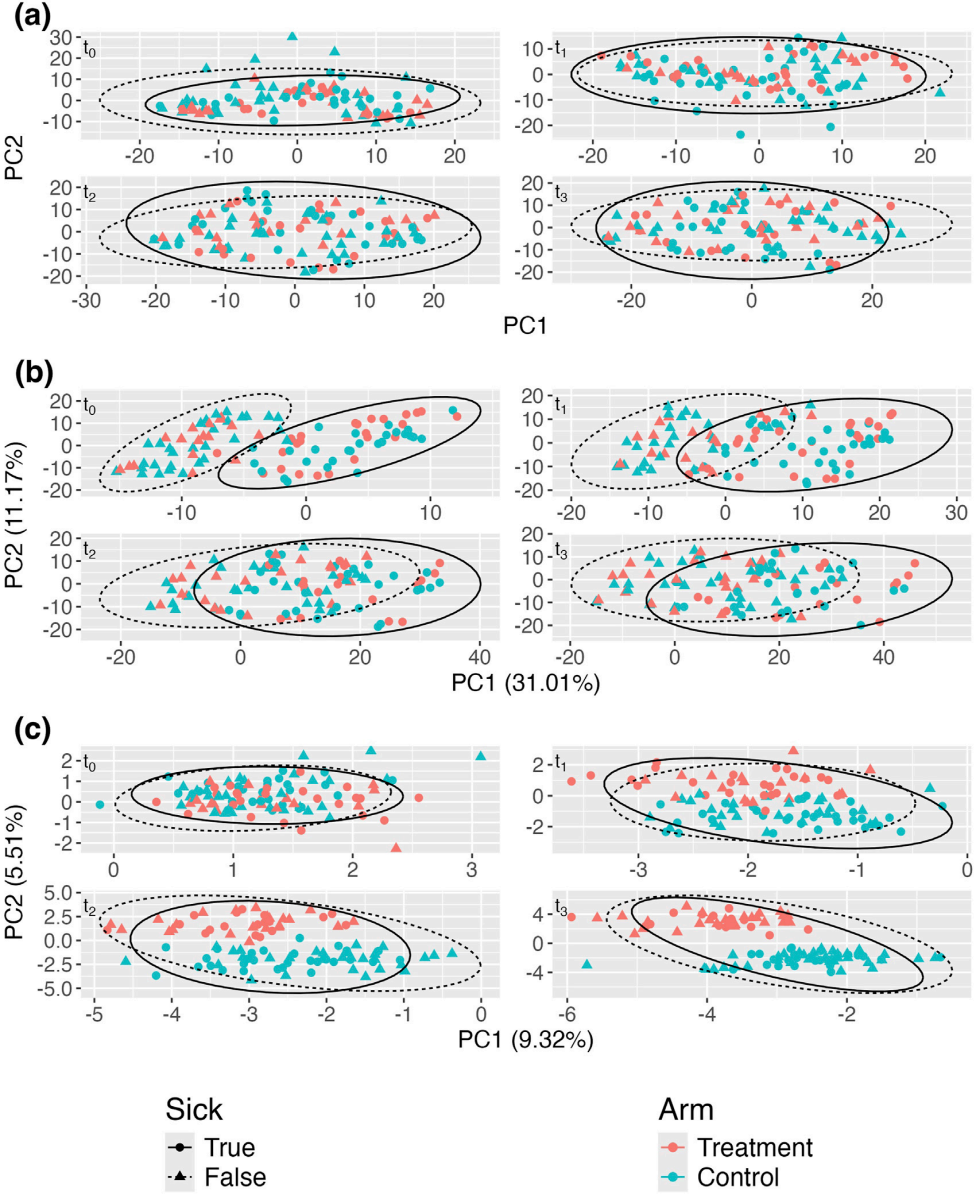
Adjusted principal coordinates analysis (aPCoA) plots of a simulated longitudinal dataset where a time-varying covariate (sickness status) confounds the treatment effect ( n=100 and m=4 time points). **(A)** aPCoA method applied to cross-sectional data, adjusting for sickness status in a linear model and stratifying by time point; **(B)** aPCoA approach adjusting solely for sickness status across all time points; **(C)** proposed aPCoA method for repeated measures, incorporating adjustment for sickness status as a fixed effect, along with a per-subject random intercept as a random effect in a linear mixed model. Points are colored by treatment group and distinguished by shape based on sickness status.

Figure 3A displays the standard aPCoA plot, stratified by time. Similar to the previous section, the treatment effect is difficult to discern, and no distinct patterns emerge, indicating that the variability in the microbiome profiles is largely dominated by other factors.

In Figure 3B, despite adjusting for sickness status as a fixed effect, the variability continues to be primarily driven by sickness. Due to the impact of repeated measures, the plot still propagates the variability associated with sickness status, further obscuring the treatment effect. However, Figure 3C reveals a much clearer distinction between treatment and control groups after adjusting for sickness status and person-specific random intercepts. This adjustment effectively isolates the treatment effect, demonstrating its influence more distinctly in the data. The comparison between these plots underscores the importance of incorporating random effects to account for confounding variables, like sickness status, to accurately visualize treatment effects in longitudinal studies.

#### 3.1.3 Hierarchical structure obscuring the effect of a time-invariant covariate effect

**FIGURE 4:**
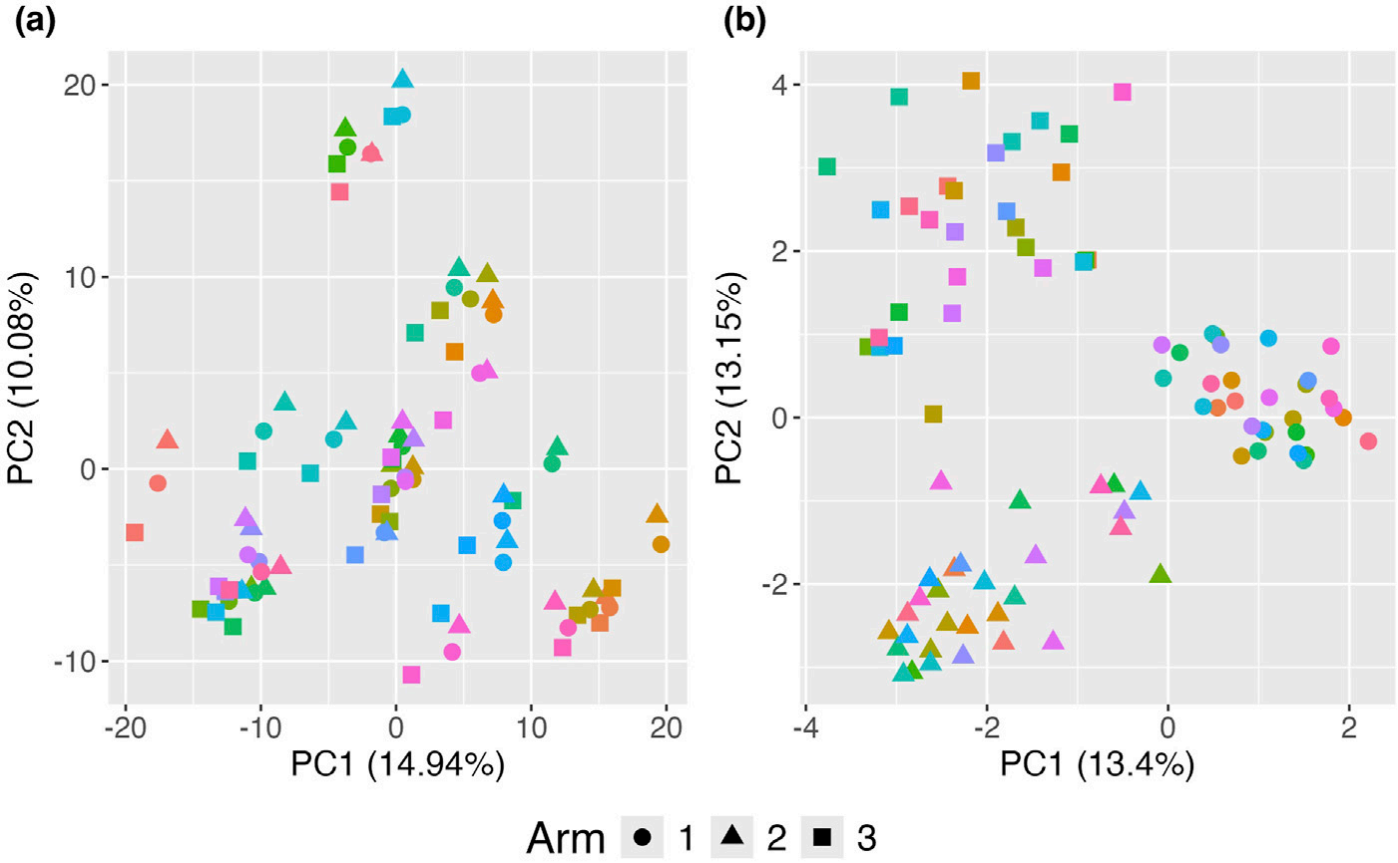
Principal Coordinates Analysis (PCoA) plots illustrating the impact of clustering on the visualization of treatment effects. **(A)** Standard PCoA plot without any adjustments, showing microbiome profiles without accounting for familial clustering. The variability due to clustering obscures the treatment effect, making it difficult to distinguish between treatment arms. **(B)** Proposed adjusted PCoA (aPCoA) plot for repeated measures, accounting for familial clustering. The treatment effect becomes clearer, with distinct separation between treatment arms, emphasizing the importance of adjusting for clustering in the analysis. Different families are represented by different colors, and treatment arms are indicated by different shapes.

The standard PCoA plot and aPCoA for repeated measures are shown in Figure 4. When using standard PCoA, the clustering effect due to family membership is not removed, making it challenging to visualize the treatment arm effect, as subjects cluster by family. However, using the proposed approach effectively removes the cluster effect, revealing clear segregation by treatment arm.

While this example focuses on a scenario where the primary interest is in the treatment effect, there may be situations where understanding clusters is of greater importance. In such cases, not adjusting for clustering might better reveal the variability of interest. Moreover, in such situations, aPCoA still allows for the option to adjust for other variables that may be less relevant.

### 3.2 MsFLASH data

We applied our proposed framework to data on 126 subjects from the Menopause Strategies: Finding Lasting Answers for Symptoms and Health (MsFLASH) Vaginal Health Trial. The trial aimed to identify microbial, immune, or metabolic markers associated with response to topical treatment for postmenopausal symptoms of vaginal discomfort. Over the course of a 12-week randomized trial, postmenopausal women were randomly assigned to a vaginal discomfort treatment of vaginal estradiol tablet plus placebo gel (arm 1), vaginal moisturizing gel plus placebo tablet (arm 2), or placebo gel and tablet (arm 3). Investigators profiled vaginal microbiota samples taken at 0, 4, and 12 weeks via 16S ribosomal RNA gene sequencing (Mitchell et al., 2021).

**FIGURE 5:**
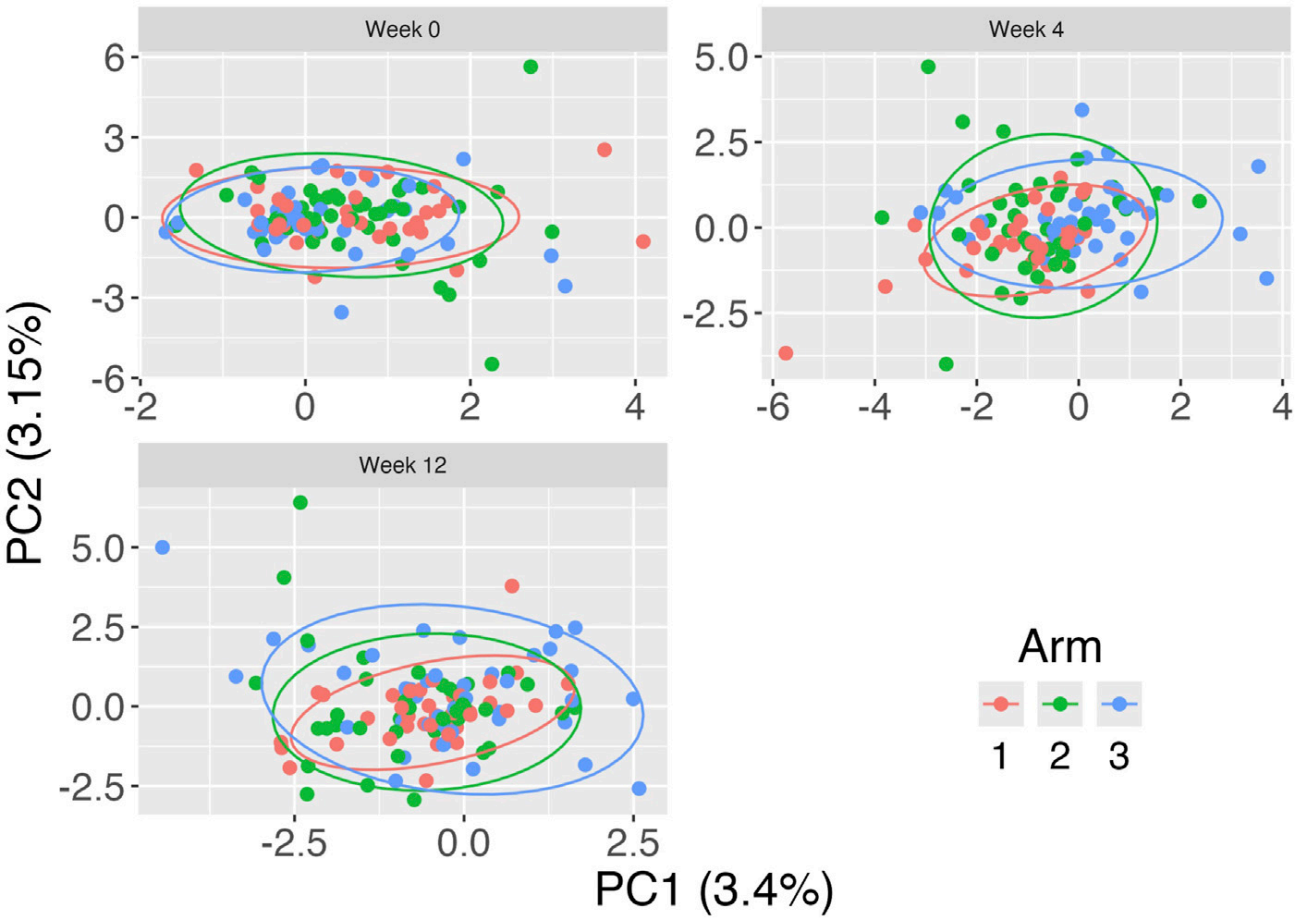
Adjusted Principal Coordinates Analysis (aPCoA) plot for repeated measures of vaginal microbiome profiles from the MsFLASH trial, evaluating the impact of vaginal estradiol tablets and/or vaginal moisturizing gel at 0, 4, and 12 weeks. Points are colored by treatment arm: vaginal estradiol tablet plus placebo gel (Arm 1), vaginal moisturizing gel plus placebo tablet (Arm 2), and placebo gel and tablet (Arm 3).

To visualize the data, we restricted our analysis to patients who had complete data measured at all three visits. We excluded taxa with zero counts across all patient measurements, resulting in 373 taxa. We used our proposed strategy with Aitchison distance, age at enrollment as a fixed effect in the LMM and included a random intercept for each subject. Adjusted PCoA plots using standardized residuals are show in Figure 5.

At Week 0, the clusters of data points for all groups overlap substantially, indicating a similar microbiome composition among the groups at the baseline. By Week 4, the data points start to show more separation, particularly for arm 3, although there is still considerable overlap among the groups. At Week 12, the separation between groups becomes more pronounced, especially for the arms 2 and 3, suggesting that microbiome composition changes over time are treatment-dependent.

Overall, the aPCoA plot indicates that while there are pre-treatment similarities in microbiome composition among the groups (which is as expected since subjects had not yet been treated), distinct changes emerge over the 12-week period, highlighting the temporal dynamics of the vaginal microbiome in response to the treatments.

### 3.3 DIABIMMUNE study

We further applied the proposed framework to the data from the DIABIMMUNE study. The study examined the gut microbiome of 39 children via 16S rRNA sequencing of stool samples and clinical information during their first 3 years of life (Yassour et al., 2016). Microbiome data were collected from participants aged between 40 and 1,105 days. Each participant underwent 16 to 32 repeated microbiome assessments, with a median of 26 measurements per individual, resulting in a total of 1,018 observations across the study. We excluded taxa that had zero counts for all measurements, leaving us with 178 taxa. We performed the steps as in 2.2 on all observations, but for the visualization in step 7, we restricted attention to the observations that occurred during the first 200 days of life.

As earlier, we used Aitchison distance and in our procedure retained the top ℓ=58 PCs, explaining approximately 90.30% of the variability. Each PC was regressed on an intercept as a fixed effect and a random intercept for each subject and standardized residuals were calculated.

**FIGURE 6:**
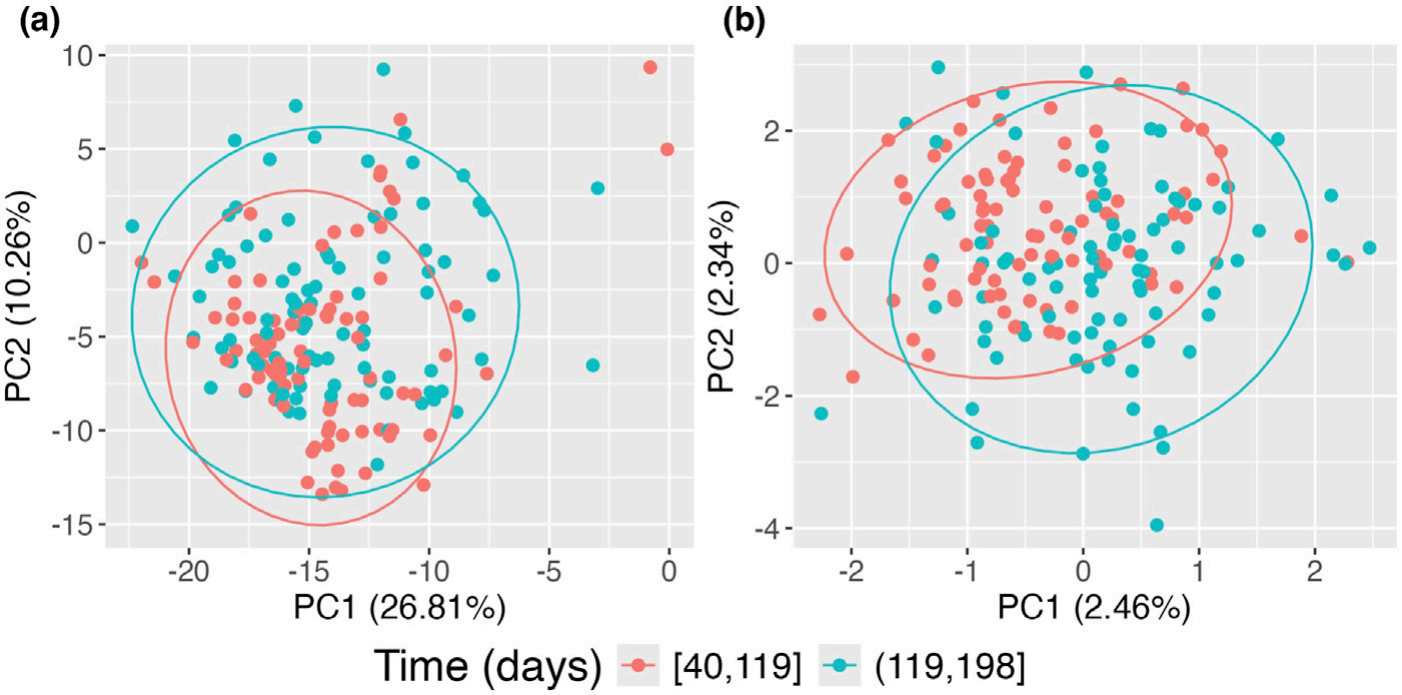
Principal Coordinates Analysis (PCoA) plots of DIABIMMUNE data restricted to the first 200 days of life. **(A)** Standard PCoA plot; **(B)** adjusted PCoA approach for repeated measures. Points are colored according to time of observation.

In Figure 6, panel (a) presents the data without accounting for repeated measurements on subjects using the standard PCoA approach, where the two time groups exhibit significant overlap. In contrast, panel (b) shows the data when repeated measurements on subjects are considered using the proposed approach. Compared to panel (a), clear separation emerges, with the earlier time group forming a tighter cluster and the later time group exhibiting a broader spread. This indicates that microbiome compositions in this sample evolve over time. However, this evolution is obscured when repeated measurements are not accounted for, as seen in the standard PCoA approach, where points from the same subjects tend to cluster together.

The results of the proposed kernel-based aPCoA analysis revealed clear temporal patterns. We also performed supplementary comparisons using CLR-transformed aPCoA, and the results are provided in the Supplementary Material for further insight on the differences between these approaches.

## 4 Discussion

While PCoA is useful for visualizing microbiome data, it is challenging to visualize data from longitudinal and clustered measures due to obscuring variables and inherent clustering. Following the approach of adjusted PCoA for cross-sectional data, we presented a general strategy for adjusting out variables that may obscure effects within a linear mixed model framework. Our results demonstrate the importance of accounting for repeated measures in microbiome studies. While traditional PCoA and aPCoA provides useful insights into microbiome structure for cross-sectional data, it does not account for the correlations between repeated samples from the same subjects, which can obscure the temporal dynamics and subject-level effects. By incorporating linear mixed models to adjust for these correlations, our approach provides more accurate visualizations of temporal patterns and treatment effects, allowing researchers to more effectively explore how microbiome profiles evolve over time. This method is particularly valuable for longitudinal studies, where repeated measures are common and require careful handling to avoid biased results.

Although we presented some possible cases, we emphasize that no single approach is universally appropriate for all situations. One must carefully consider which variables to adjust and whether to reduce effects of clustering, depending on the study and analytic objectives. In general, we suggest constructing unadjusted plots, over time, to identify major variables that may be making the visualization more challenging. These can then be included as fixed and random effects as appropriate. Similarly, visualization of clustering effects can be used to ascertain whether adjustment would be helpful.

One facet of our approach is that the proportion of variability explained by variables may change after adjustment. The proportion explained by variables of interest could increase or decrease depending on the variable and the specific study, but in either case, one should avoid testing associations between beta-diversity and variables of interest [e.g., permanova (Anderson, 2001; Zhao et al., 2015)] using the corrected PCs or the corrected similarity matrices.

Our proposed strategy relies heavily on linear mixed models and accordingly, is similarly constrained by the limitations of the LMM. In particular, LMMs typically assume normality of the outcomes (in this case the PCs rather than the taxonomic abundances) and random effects, and we have focused only on modeling linear main effects of the variables. Similarly, sufficiently large sample size is necessary to fit these models stably. While it is possible to consider more sophisticated models that mitigate these limitations within the context of LMMs, we emphasize that our focus is on visualization rather than formal inference. Therefore, deviations from the usual distributional assumptions do not affect the statistical validity of the visualization. The requirement for sufficient sample size is important, but as longitudinal microbiome studies continue to get larger, this issue will be resolved. However, larger sample sizes will further emphasize the need for adjusted procedures like ours to uncover more subtle signals.

Our proposed kernel-based aPCoA method provided more distinct separation and clearer visualization of temporal patterns compared to the simpler CLR-transformed aPCoA approach. While the results from the CLR-transformed method were broadly consistent, the visualizations were less distinct and failed to capture some of the more subtle temporal dynamics. These supplementary findings, detailed in the Supplementary Material, highlight the advantages of the kernel-based method in handling repeated measures and covariate adjustment, despite the potential trade-off in methodological complexity. The kernel-based approach ultimately offers better results in handling repeated measures and covariate adjustment, making it the superior choice in more complex datasets.

An important aspect of our approach is the preprocessing of input microbiome data, particularly the decision to normalize the data. The use of the centered log-ratio (CLR) transformation, paired with the Aitchison kernel matrix, proved effective in handling compositional microbiome data and addressing issues related to differences in sequencing depth. Normalization via CLR ensured that the data were comparable across samples, leading to more meaningful visualizations. However, users should carefully consider their choice of transformation and kernel matrix, as these decisions can significantly impact the results of the analysis.

In particular, when we constructed a Bray-Curtis kernel matrix using relative abundance data, the resulting visualizations were less meaningful, likely due to the kernel’s inability to appropriately account for the compositional nature of the data. This underscores the importance of choosing a kernel matrix and data transformation that align with the underlying structure of microbiome data. For studies where relative abundance is the focus or where compositionality is less of a concern, a Bray-Curtis kernel matrix may be suitable. However, for compositional data, the Aitchison kernel matrix, combined with CLR, remains the preferred choice due to its ability to properly handle the relative nature of microbiome datasets. In addition to these choices, some kernel matrices, such as UniFrac, account for phylogenetic relationships between taxa. In studies where evolutionary history plays a central role, a kernel matrix based on UniFrac could provide valuable insights by incorporating this phylogenetic information. However, for compositional data where relative abundances are of primary interest, the Aitchison kernel remains the preferred choice. Future research could further explore the impact of using different kernel matrices, such as phylogeny-aware approaches like UniFrac, to extend the applicability of our method to a broader range of microbiome studies.

Additionally, while the choice of normalization and kernel matrix plays a key role in the success of the method, the performance of our approach remains robust as long as appropriate preprocessing steps are applied. In future studies, exploring how different transformations and kernel matrices impact the visualization of longitudinal and repeated measures microbiome data could further enhance the applicability of this method across a wider range of microbiome studies.

## Data Availability

The MsFLASH vaginal microbiota sequences have been deposited in the National Center for Biotechnology Information (NCBI) Sequence Read Archive (SRA), accession PRJNA788936. The DIABIMMUNE data have been deposited in NCBI BioProject, accession PRJNA290381.
