# Supplementary material for: Enhanced visualization of microbiome data in repeated measures designs
Source: Front Genet. 2024 Nov 15;15:1480972. doi: 10.3389/fgene.2024.1480972 (PMC11604720; doi:10.3389/fgene.2024.1480972)
Supplement: Supplementary file 1 [file DataSheet1.pdf]

Supplementary Materials

S1. Comparison of aPCoA on CLR-Transformed Data

We conducted a comparison between the results of adjusted PCoA (aPCoA) on CLR-transformed data and our proposed kernel-based method. The aim of this comparison was to evaluate whether the simpler CLR-transformed approach could yield results comparable to those from the more complex kernel-based method, particularly in the context of handling repeated measures and adjusting for covariates. If the CLR-transformed approach proves equally effective, it may offer a more straightforward and simpler implementation without sacrificing the quality of the results.

In this comparison, we applied linear mixed models (LMMs) to CLR-transformed data to adjust for confounders and repeated measures, followed by PCoA on the adjusted data. The resulting plots were directly compared to those generated by our proposed method. This analysis was conducted using the dataset from the simulation described in sections 2.3.1 and 3.1.1, as well as the real data application in section 3.3. The results are shown in Figures S1 and S2.

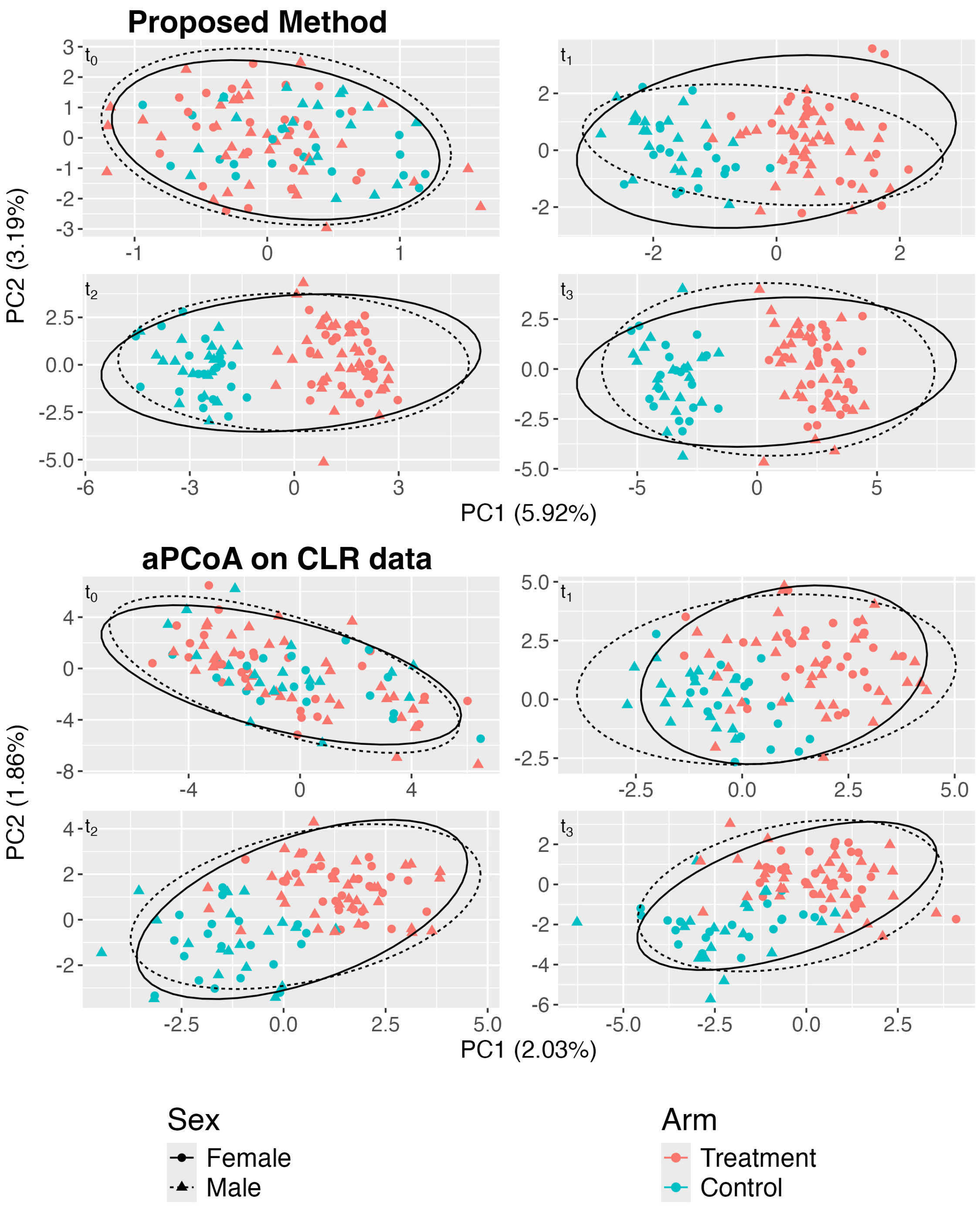

Figure S1: Comparison of the proposed kernel-based aPCoA method and aPCoA on CLR-transformed data. The top row shows the results from the proposed kernel-based aPCoA method applied to repeated measures microbiome data. The bottom row presents the results from applying aPCoA on CLR-transformed data. Data are derived from the simulation described in Sections 2.3.1 and 3.1.1.

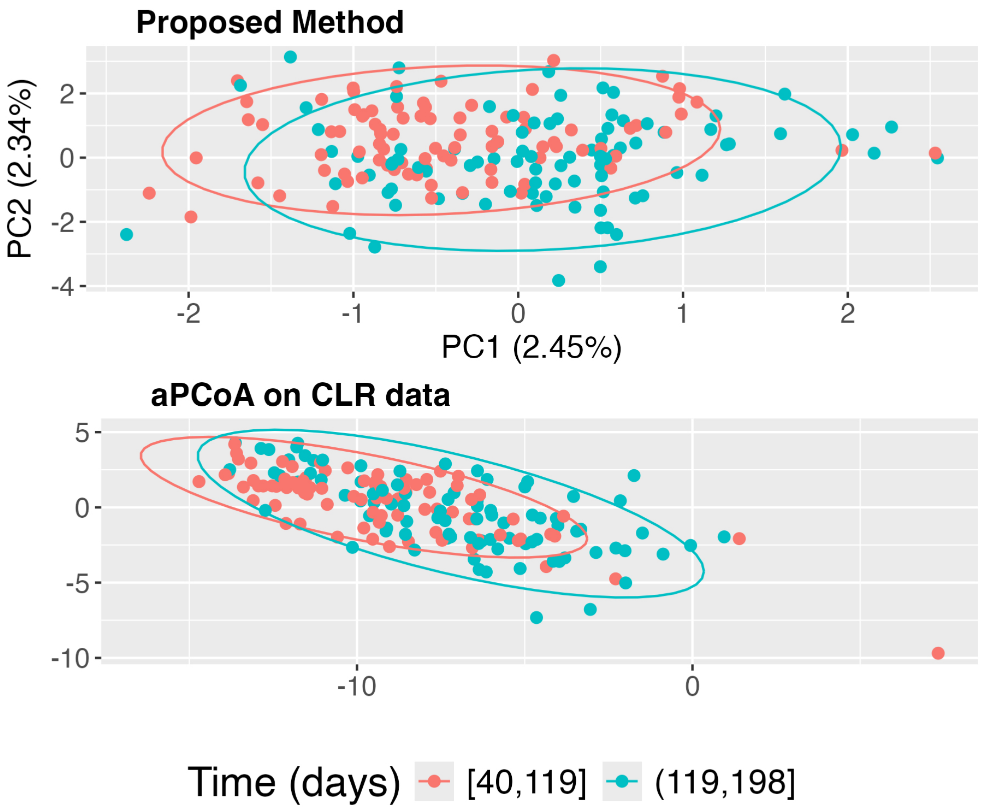

Figure S2: Comparison of the proposed kernel-based aPCoA method and aPCoA on CLR-transformed data. The top panel displays the results from the proposed kernel-based aPCoA method applied to microbiome data. The bottom panel shows the results from aPCoA on CLR-transformed data. The data correspond to the real-world application described in Section 3.3.

Figure S1 shows that, while CLR-transformed PCoA adjusts for some confounding, the temporal dynamics remain less distinct compared to our adjusted aPCoA method. In Figure S2, the CLR-transformed PCoA shows some separation between groups, but the ability to visualize temporal changes is still more limited compared to our proposed approach.

Additionally, our method may be more sensitive to global changes in microbiome composition due to its kernel-based approach, which captures the overall structure of the data more effectively than CLR-transformed PCoA. Furthermore, our method can incorporate alternative kernel matrices, such as those that account for phylogenetic relationships (e.g., UniFrac), providing flexibility for studies where phylogeny is important in the visualization. This adaptability makes our approach suitable for a wider range of microbiome data structures.

While both approaches offer valuable insights, we believe that our method more effectively addresses the complexity of longitudinal microbiome data and provides more accurate visualizations of temporal dynamics.
